# Immunity to *Cryptococcus neoformans* and *C. gattii* during cryptococcosis

**DOI:** 10.1016/j.fgb.2014.11.006

**Published:** 2015-05

**Authors:** Josie F. Gibson, Simon A. Johnston

**Affiliations:** a Department of Infection and Immunity, Medical School, University of Sheffield, S10 2RX, UK; b Bateson Centre, Department of Biomedical Sciences, University of Sheffield, S10 2TN, UK

**Keywords:** *Cryptococcus neoformans*, *Cryptococcus gattii*, Cryptococcosis, Macrophages, T cells, Immunity

## Abstract

The vast majority of infection with cryptococcal species occurs with *Cryptococcus neoformans* in the severely immunocompromised. A significant exception to this is the infections of those with apparently normal immune systems by *Cryptococcus gattii*. Susceptibility to cryptococcosis can be broadly categorised as a defect in adaptive immune responses, especially in T cell immunity. However, innate immune cells such as macrophages play a key role and are likely the primary effector cell in the killing and ultimate clearance of cryptococcal infection. In this review we discuss the current state of our understanding of how the immune system responds to cryptococcal infection in health and disease, with reference to the work communicated at the 9th International Conference on Cryptococcus and Cryptococcosis (ICCC9). We have focussed on cell mediated responses, particularly early in infection, but with the aim of presenting a broad overview of our understanding of immunity to cryptococcal infection, highlighting some recent advances and offering some perspectives on future directions.

## Highlights

• *Cryptococcus neoformans* and *C. gattii* cause infection in the immunocompromised and immunocompetent respectively.
• Compromised T cell immunity is the main predisposing factor for cryptococcal infection and cryptococcal meningitis.
• Immunity to *Cryptococcus* relies on innate immune cells coordinating adaptive responses to stimulate fungal killing.
• Differences in *C. gattii* immunity are not well studied but may result from differences in activation of inflammation.

## 1 Introduction

*Cryptococcus neoformans* and *Cryptococcus gattii* are environmental yeasts that cause opportunistic infection. The most common and serious type of cryptococcal disease is pulmonary cryptococcosis that is uncontrolled and leads to cryptococcal meningitis (CM) or meningoencephalitis. CM is almost exclusively observed in those with severely impaired immunity. Rarely other forms of cryptococcal infection are seen (e.g. cutaneous infection, peritonitis; (Christianson et al., 2003; El-Kersh et al., 2013) but this review will focus on infection and immunity to CM (Jarvis and Harrison, 2007).

*C. neoformans* infection is a leading cause of mortality within AIDS patients worldwide, particularly in sub-Saharan Africa (Park et al., 2009). In addition to HIV infected individuals, *C. neoformans* is also associated with other immunocompromised groups such as those with haematopoietic malignancy, solid transplant patients (e.g. glucosteroid therapy) and therapy for rheumatoid arthritis (e.g. anti-TNFα mAb). In contrast, *C. gattii* is capable of infecting immunocompetent individuals, most notably in the Vancouver Island outbreak (Kidd et al., 2004), and is generally absent in the immunocompromised (Chen et al., 2000; Mitchell and Perfect, 1995). There are a number of counter examples e.g. *C. gattii* infection of HIV infected individuals (Springer et al., 2014; Steele et al., 2010) and certain *C. neoformans* genotypes infecting the immunocompetent (Chau et al., 2010). *C. neoformans* is found globally and has been isolated from pigeon excrement, soil and rotting vegetables, whereas *C. gattii* is predominantly restricted to tropical and sub-tropical areas, and is associated with eucalyptus trees (Bovers et al., 2008; Voelz and May, 2010).

## 2 Overview of host immunity during cryptococcosis

In this section we introduce the broad concepts of the immune response and pathogenesis of cryptococcosis before exploring each part in greater detail in the sections below. Pulmonary cryptococcal infection will occur only if fungal cells, environmentally acquired by inhalation, are deposited deep in the lungs. After deposition within the lung, alveolar macrophages (AMs), tissue resident macrophages that will remove microorganisms and other particulates, will be the first cells to encounter the cryptococci and will respond by internalising them through phagocytosis (Goldman et al., 2001). Following phagocytosis AMs will ideally kill the microorganisms and initiate and modulate the appropriate wider immune response. This will include the release of cytokines, activation and recruitment of other immune cells and presentation of antigen.

Due to the resolving sub-clinical or mild nature of the infection in immunocompetent individuals there is limited data on the normal immune response to *C. neoformans*. Cryptococcal infections are occasionally identified due to medical examination for other symptoms or conditions. In these cases cryptococci are enclosed within granulomas (Haugen and Baker, 1954). Granulomas are a diverse group of immune structures, commonly recognised as macrophage like cell clusters surrounded by lymphocytes. Their primary function is to control and remove infections but are also seen in a number of immune diseases (Rose et al., 2014). Cryptococcal granulomas are compact, with macrophages, multinucleated giant cells (both containing intracellular yeasts) and CD4+ lymphocytes (Shibuya et al., 2005, 2002). These granulomas will typically resolve over a period of weeks or months without clinical intervention. Evidence of childhood infection (cryptococcal antibody profile increasing with age) suggests latent infection, or repeated infection (Goldman et al., 2001). In addition, decline of immune efficiency as a trigger for the reemergence of a dormant cryptococcal infection, potentially suggests that cryptococci are able to escape degradation and remain within an individual for long periods of time (Garcia-Hermoso et al., 1999).

It is clear from both the clinic and experimental models of cryptococcosis that T cell responses are key to the control of cryptococcal infection. The major defect in the patient groups at risk of developing cryptococcal meningitis is in the CD4+ T helper system. Therefore, a very different response in the lung is seen in individuals with AIDS. Loosely aggregated giant cells are seen but cryptococci are typically extracellular and there is significant proliferation in the alveolar spaces (Shibuya et al., 2005, 2002). If cryptococcal infection is not controlled in the lungs then it will disseminate throughout the body, with particular preference for the central nervous system (CNS). Infection of the CNS results in life threatening meningitis and/or meningoencephalitis, with very high mortality without treatment (Bovers et al., 2008; Mitchell and Perfect, 1995). The route and mechanism of spread to the CNS has not been directly demonstrated, however, it is likely through the blood as the lymphatic system has no access to the brain (Liu et al., 2012; Iliff and Nedergaard, 2013).

## 3 The anatomical and upper respiratory defences of the lungs and the infectious propagule of cryptococcosis

The human respiratory system is highly adapted to removing air borne contaminants while maintaining airflow for efficient gas exchange. The combination of airflow turbulence, the physical epithelial mucosal barrier, the trapping of particulates in mucus and their subsequent removal via mucociliary transport, results in the nose and upper airways largely preventing entry of particles greater than 2 μm (Nicod, 2005). As the lungs are able to exclude particles greater than 2 μm the infectious agent in cryptococcal infection is hypothesised to be the spore or desiccated yeast cell as they will be small enough to travel deep in the lower respiratory tract (Botts and Hull, 2010; Velagapudi et al., 2009).

Furthermore, the airways are highly populated by dendritic cells (DCs) that clear microorganisms through phagocytosis, and there are large quantities of the immunoglobin IgA that function to bind toxin and viral particles as well as impede bacterial invasion of epithelial cells (Nicod, 2005). While upper airway DCs may play a role in preventing cryptococcal yeast cells from getting deep within the lungs, there is no evidence that airway IgA is required for prevention of cryptococcal infection. However, it is intriguing to speculate whether upper airway DCs may represent an alternative route of primary infection for the immunocompromised.

## 4 Lung surfactant as a barrier to cryptococcal infection

The inner surface of alveoli are covered in a film of surfactant that functions to ensure the alveoli in the lung are capable of full re-inflation, through maintenance of surface tension (Schurch et al., 1992). Surfactants are also known to play a role in the lung immune defence with particles deposited within the lung being pulled towards the alveoli wall by the surfactant promoting the interaction of pathogens and surfactant proteins (Schurch et al., 1992). Surfactant proteins SP-A and SP-D are members of the immune collectins. They bind to pathogens and promote macrophage and neutrophil immune function by opsonising pathogens and increasing phagocytosis (Kishore et al., 2005; Wright, 2004). Both SP-A and SP-D preferentially bind to pathogens over host cells through favoured binding to pathogen associated molecules e.g. mannose (Kishore et al., 2005).

SP-A and SP-D are known to bind viral, bacterial and fungal pathogens, including *C. neoformans* (Schelenz et al., 1995). SP-A and SP-D bind both acapsular and encapsulated *C. neoformans* but the effect of binding differs. SP-A bound to encapsulated yeast was not beneficial to the host, as a murine SP-A knockout model showed no difference in survival to wild type (Giles et al., 2007). This suggests that SP-A will have little effect once *C. neoformans* has induced capsule synthesis and may be one mechanism by which the cryptococcal capsule promotes virulence through blocking the action of SP-A. Similarly, SP-D can bind both acapsular and encapsulated yeast but there is only increased phagocytosis of acapsular yeast *in vivo* (Geunes-Boyer et al., 2009). There is evidence that SP-D promotes virulence of encapsulated *C. neoformans* as SP-D knock-out mice were better able to control cryptococcal infection than wild-type mice (Geunes-Boyer et al., 2012, 2009). Two possible explanations for this are that SP-D protects cryptococci from macrophage mediated degradation (so far only demonstrated *in vitro*) and that SP-D promotes the recruitment of eosinophils that may be detrimental to the control and clearance of pulmonary cryptococcosis (Holmer et al., 2014).

## 5 Phagocytosis of cryptococci by macrophages and dendritic cells

If spores or yeast cells enter the alveolar spaces they will encounter alveolar macrophages (AM). There are a number of different subsets of both macrophages (and DCs) that are present in the lungs (Guilliams et al., 2013), but for the purpose of this review we will refer generally to macrophages and DCs.

Macrophage control of cryptococci is essential early in infection with depletion of AMs in the lungs of rats (a normally resistant host) leading to high fungal burden and dissemination following intratracheal infection (Shao et al., 2005). Interestingly, it has been recently demonstrated that phagocytosis *in vitro* can be associated with fungal burden in a patient cohort, where increased uptake by macrophages was associated with poor prognosis (Sabiiti et al., seminar presentation 9.5, ICCC9; Sabiiti et al., 2014). However, both the mechanism governing the difference in phagocytic uptake of these strains, and the point during infection where the phagocytosis phenotype is critical in human infection, are yet to be identified. Furthermore, in mice deficient in macrophages and DCs there were increased numbers of PMNs and B cells in response to cryptococcal infection. Neutrophils and B cells alone were unable to control the infection and their continued presence and activation is potentially highly damaging to the host (Osterholzer et al., 2009).

The uptake of cryptococci by phagocytes *in vitro* is enhanced by the presence of antibody and/or complement as opsonin. Opsonin provides an adapter between receptors on phagocytes and the surface of the microorganism. The Fc receptors on macrophages and dendritic cells bind to the constant region of antibody and the binding of a sufficient cluster of Fc receptors activates actin mediated uptake of the microorganism. Fc receptor signalling does not only lead to uptake but also can modulate the activation and signalling state of the phagocyte. Recently, a polymorphism in the antibody binding receptor, Fc gamma receptor 3A (FcGR3A), has been identified as a risk factor for male, HIV infected individuals (Rohatgi et al., 2013). Increased IgG complexes found in HIV infected individuals, combined with the FcGR3A polymorphism having a higher affinity for these immune complexes, may result in increased phagocytosis, and therefore greater intracellular proliferation and spread within the host. A second polymorphism has also been identified in FcγRIIB in a non HIV cryptococcal meningitis cohort (Zhu et al., seminar presentation 3.1, ICCC9; Hu et al., 2012).

Similarly to Fc mediated uptake, complement binds to a heterodimer of integrin receptors (CR3; CD11b/CD18) and activates actin mediated phagocytosis. The complement cascade completes three key roles during pathogen infection: Opsonisation, direct killing of pathogens and recruitment of inflammatory cells. The complement cascade converges with the production of C3 convertase, and subsequent creation of C3b, via three main pathways; classical, lectin and alternative, which in turn lead to production of C5a and C5b. The alternative pathway mediates the role of complement protein in host defence against *C. neoformans*, although nonencapsulated cryptococci are able to activate the classical pathway (Kozel et al., 1991; Mershon-Shier et al., 2011). Cryptococcal capsule accumulates C3 via the alternative pathway and deposition of C3 near the surface is needed for efficient phagocytosis. However, if C3 is evenly distributed throughout the capsule increased volume of capsular polysaccharide will reduce the amount on the surface resulting in reduced complement mediated phagocytosis (Gates and Kozel, 2006; Zaragoza et al., 2003). To add to this, recently a cryptococcal hydrolytic enzyme, lactonohydrolase (LHC1), was shown to combat the complement cascade through inducing capsular changes which reduces C3 deposition on the capsule, a *lhc1* mutant that had increased C3 deposition and decreased virulence (Park et al., 2014). Another complement protein, C5 is also implicated, with mice strains more susceptible to cryptococcal infection when C5 was not present in their plasma (Rhodes et al., 1980).

Phagocyte pattern recognition receptors (PRRs) are important in phagocytosis both in combination and independent of opsonic recognition. PRR bind to specific components of microbes, usually on their surface (exceptions being those receptors that bind to nucleic acid) and although often implicated in phagocytosis, are not all direct activators of pathogen internalisation. Mannose and the beta glucan are the primary pathogen recognition motifs for PRR mediated recognition and uptake of cryptococci. However, the polysaccharide capsule will interfere sterically and chemically with the recognition of these motifs. As the capsule is likely to be very small or absent in the infectious propagule, cryptococcal mannoprotein and beta glucan will be determinants in early interactions in the lung. In support of this, mannose receptor knockout mice are more susceptible to infection and show reduced CD4+ T cell proliferation in response to cryptococcal mannoprotein. Uptake of cryptococcal mannoprotein by DCs was not affected most likely through alternative sampling mechanisms such as antigen presentation from the phagosome or environmental sampling via macropinocytosis (Dan et al., 2008).

Recognition via scavenger receptor A (SR-A) (classically a receptor for low-density lipoprotein but which also interacts with a number of pathogens (Mukhopadhyay and Gordon, 2004)) appears to promote a Th2 immune profile. The SR-A deficient mouse demonstrated a better host response in terms of fungal clearance coupled with decreased IL4 and IL13 production (Qiu et al., 2013). In contrast, the class B scavenger receptors CD36 and SCARF may be a requirement for pro inflammatory Il-1beta, as the absence of CD36 resulted in a modest reduction in survival in the susceptible C57BL/6 stain after acute intravenous infection (Means et al., 2009). These two studies represent the best evidence that the specific phagocytic receptor involved in uptake of cryptococci into macrophages may directly regulate immune signalling and therefore the outcome of infection. This is, potentially, a highly significant area of *Cryptococcus* research but there are still fundamental gaps in our understanding, for example, a direct demonstration that scavenger receptors mediate the uptake of cryptococci *in vivo*.

Toll like receptors (TLRs) are a highly conserved group of PRRs conserved across vertebrates (O’Neill, 2004). The TLR9 deficient mouse showed reduced CCL7 production (Cheng et al., 2014), and restoration of CCL7 over the first week of infection relieved the associated suppression of interferon gamma and recruited DC numbers (Qiu et al., 2012). However, restoration of CCL7 was insufficient to suppress increased fungal burden. How TLR9 is activated by cryptococci when it classically responds to unmethylated CpG dinucleotides in prokaryotes is not known, although activation of TLR9 by other eukaryotic pathogens has been described (Nakamura et al., 2008). The roles of TLR2 and TLR4 have been investigated but currently their role appears minor in respect to overall control of cryptococcal infection (Biondo et al., 2005; Nakamura et al., 2006). Treatment of microglia cells with agonists to TLRs 2, 3, 4 and 9 increased pro inflammatory cytokines in response to cryptococci but the physiological relevance of this is not clear (Redlich et al., 2013).

Recently, another class of PRR, related to the TLRs, the intracellular NOD like receptors have been implicated in cryptococcal infection. Acapsular cryptococcal cells were able to activate NLRP3, requiring direct cell wall contact. Mice deficient in *Nlrp3* had reduced infiltration of leukocytes into the lung and increased fungal burden. Cryptococcal capsule is able to block this interaction again suggesting that PRR interactions will be especially important early in infection (Meng, seminar presentation 7.1, ICCC9; Guo et al., 2014).

## 6 Intracellular cryptococci in macrophages

After phagocytosis, the newly formed phagosome undergoes a series of vesicle fusion and maturation steps to generate a highly antimicrobial environment. Many intracellular pathogens, for example *Mycobacterium tuberculosis*, interfere with the maturation of the phagosome. In contrast, cryptococci apparently do not evade the acidified phagosome, as they survive within Lamp1 positive vesicles at low pH (Johnston and May, 2010; Levitz et al., 1999; Vandal et al., 2009).

In addition to the classical phagosome maturation pathway, the cell content recycling pathway of autophagy has been implicated in the response to a number of pathogens particularly those that can escape the phagosome (Cemma and Brumell, 2012). Components of the autophagy pathway were first implicated in the handling of cryptococci in a *Drosophila* S2 cell RNAi screen (Qin et al., 2011) and the autophagy effector LC3 has been observed associated with cryptococcal phagosomes (Nicola et al., 2012). Conditional macrophage ATG5 deficient mice showed no difference in survival, although histology of lung lesions showed reduced inflammation (Nicola et al., 2012). There are multiple routes into activation of autophagy and the lack of strong phenotype may be explained by compensation of alternative autophagy activation pathways (Cemma and Brumell, 2012).

*C. neoformans* is able to proliferate within the macrophage phagosome (Tucker and Casadevall, 2002; Voelz et al., 2009). This suggests that *C. neoformans* is able to inhabit the macrophage as a protective niche within the host. *C. neoformans* is also able to travel from one macrophage to another, whereby the receiving macrophage accepts the cryptococcal cell in an actin dependant manner (Alvarez and Casadevall, 2007; Ma et al., 2007). This is a rare event *in vitro* that has yet to be observed *in vivo*. The cause of this phenomenon, and if it benefits the host or the pathogen is unknown. The receiving cell may be assisting a potentially moribund macrophage or lateral transfer between macrophages allows evasion of extracellular host immune responses by cryptococci.

Cryptococci can escape macrophages by lysis or through expulsion. The mechanism of lysis is unknown. In some cases this appears to result from intracellular replication leading to large numbers of intraphagosomal cryptococci causing rupture of the host cell membrane (Tucker and Casadevall, 2002). Several studies have identified that the cryptococcal phagosome is permeabilised, but by two different mechanisms: Pores to the cytoplasm (Tucker and Casadevall, 2002) and extracellular phagosome emptying (Carnell et al., 2011; Johnston and May, 2010). Expulsion of cryptococci is described as a non-lytic escape from macrophages via an actin independent exocytosis (Alvarez and Casadevall, 2006; Johnston and May, 2010; Ma et al., 2006). Cryptococci leave the macrophage without causing damage to the host cell. Again as with lateral transfer the ultimate beneficiary is unknown.

The direct role of all these macrophage parasitic phenomena *in vitro* has yet to be demonstrated in pathogenesis but they support the hypothesis that macrophages are a controlling measure that require wider host immunity to clear cryptococci. In addition, macrophages may act as Trojan horses for the dissemination of cryptococci, a model that has been elegantly supported by showing that mice infected intravenously with cryptococci within macrophages showed much faster dissemination than free yeast (Charlier et al., 2009).

## 7 Dendritic cells

As professional antigen presenting cells, dendritic cells lead to activation of the adaptive immune system, which generates a specific immune response to infection, with clear differences in the roles of DCs and macrophages. However, it should be noted that there may be significant overlap in function and in many cases the relative contributions of each cell type (and sub-types) in the responses to cryptococci are not known.

DCs appear to be the primary route by which cryptococcal antigen is presented to stimulate T cells, although a previous study had suggested that AMs might also be able to stimulate T cells via cryptococcal antigen presentation (Mansour et al., 2006; Syme and Spurrell, 2002; Vecchiarelli et al., 1994)). Like other phagocytes, dendritic cells require opsonins to enable high levels of phagocytosis of *C. neoformans* with contribution from the mannose receptor. Both complement and anticapsular antibody have been shown to increase cryptococcal phagocytosis in human dendritic cells, resulting in TNFα secretion and dendritic cell activation (Syme and Spurrell, 2002; Kelly et al., 2005). Once phagocytosed, cryptococci are degraded through oxidative and non-oxidative mechanisms, after passage through lysosomes. One interesting example is a non-enzymatic role for cathepsin B, a degradative enzyme found in the lysosomal compartment, whereby osmotic lysis leads to rupture of the cell wall (Hole et al., 2012). Degraded components are then loaded onto major histocompatibility complex class II molecules ready for T lymphocyte and ultimately, adaptive immune system activation (Wozniak et al., 2006). This has been demonstrated *in vivo* with a murine model, where pulmonary cryptococcal infection led to rapid death of mice in which dendritic cells in addition to alveolar macrophages were depleted, due to inability to control the infection (Wozniak et al., 2006).

As important as their initial interaction with pathogens as is the role of DCs and macrophages in recruitment and coordination of the wider immune response. Much of this stems from the production of cytokines and chemokines, and the influence these have on the recruitment and activation of other cell types. However, AMs must first initiate this process first through recruitment of macrophages (and monocytes) and then DCs. The production of macrophage chemotactic protein 1, after phagocytosis of cryptococci in a rat model of chronic pulmonary cryptococcosis, demonstrates an important step in the initiation of a successful granulomatous response (He et al., 2003). Similarly, CCR2 is required for the recruitment of DCs in response to cryptococcal infection and in CCR2 deficient mice the inflammatory T cell immune response is immediately disrupted (Osterholzer et al., 2008). Interestingly, in absence of DCs, in CCR2 deficient mice, there was an increase in the number of macrophages observed (Osterholzer et al., 2008). The increase in macrophage numbers may be a direct response to the failure to transition to the next stage of immune control (i.e. a failure to dampen the macrophage recruitment signal) or may be macrophages responding directly to maintain control of the cryptococcal infection.

## 8 Neutrophils

Neutrophils, like macrophages and dendritic cells, capture and degrade pathogens and have a particular role in the initiation of inflammation, in response to infection. Thus, neutrophils may be expected to play an important role in cryptococcal clearance. However, there is little evidence suggesting that neutrophils are necessary for control of cryptococcosis as there is no association between diseases of the neutrophil (e.g. chronic granulomatous disease, neutropenia) alone being a predisposing factor for cryptococcosis, but only in conjunction with additional immune deficiency (e.g. (Hirai et al., 2011)). Mouse studies support this conclusion, as although neutrophils are able to phagocytose and degrade *C. neoformans*, after neutrophil depletion the fungal burden is not enhanced (Mednick et al., 2003; Wozniak et al., 2012). Furthermore, in mice depleted of neutrophils, survival was improved, possible through an up-regulation of both Th1 and Th2 cytokines (Mednick et al., 2003). As a Th1 profile is thought to be essential in cryptococcal clearance, the combined effect of both profiles may lead to improved cellular clearance with minimised host damage, perhaps because, in normal infection, neutrophilic responses may actually be detrimental in terms of organising a Th1 vs Th2 skew.

Despite a potential key role in orchestrating the type of immune response, neutrophils are primarily active in their use of oxidative and non-oxidative mechanisms to combat and degrade microbes. Neutrophils release reactive oxidative species and their intermediates (hydroxyl and hypochlorite ions) on encountering cryptococci. However, *C. neoformans* is able to counteract this host defence, through production of mannitol, which acts as a scavenger for these reactive oxygen intermediates, thus reducing their potential for cellular damage (Chaturvedi et al., 1996). Similarly, melanised cryptococci are also resistant to this neutrophil attack (Qureshi et al., 2011). A non-oxidative host defence delivered by neutrophils is through production of proteins found within their other granule types. These proteins, for example defensins (that are not present in mouse neutrophils (Eisenhauer and Lehrer, 1992)) exert a degradative effect on cryptococcal cells, in addition to oxidative mechanisms (Mambula et al., 2000). These anti-cryptococcal defences highlight how neutrophils are able to combat and degrade invading cryptococci, despite fungal burden not being neutrophil dependant.

Neutrophils are activated by contact with cryptococcal components, for example the dose dependent release of pro-inflammatory cytokines in response to cryptococcal capsule (Retini et al., 1996). This indicates a host mechanism that is capable of using the cryptococcal capsule to recognise infection and may lead to further neutrophil recruitment. Conversely, neutrophils are attracted to sites of infection, and CD18 is an adhesion molecule involved in this step. It is suggested that cryptococcal capsule molecules are able to bind and functionally inhibit CD18, leading to impaired attraction of neutrophils to the site of infection (Dong and Murphy, 1997). This may indicate that *C. neoformans* actually halts the recruitment of neutrophils, potentially to avoid the release of pro inflammatory cytokines.

Individuals with late stage HIV infection do not show differences in the number neutrophils but specific functional defects have been described. For example, reduced expression of CD88, a C5a receptor, results in a decrease in IL-8 expression in response to cryptococcal infection and a subsequently reduced inflammatory response (Monari and Casadevall, 1999).

## 9 Eosinophils

Eosinophils are granulocytes that primarily function by releasing cytotoxic granule proteins (Blanchard and Rothenberg, 2009; Feldmesser et al., 1997). However, although not professional phagocytes, eosinophils are capable of phagocytosis. In a rat cryptococcal model, eosinophils where shown to phagocytose cryptococci, become activated and produce a Th1 profile, indicating a role of activating the adaptive immune system, shown with an increase in MHC molecules (Garro et al., 2010). Furthermore, the interaction with cryptococci down regulated eosinophil NO and H_2_O_2_ production that may help to prolong eosinophil life, and therefore their efficacy during infection to act as an APC. Eosinophils activated by cryptococci were observed to migrate to the spleen and mesenteric lymph nodes and stimulate specific T cell and B cell proliferation (Garro et al., 2011). This suggests a positive role of eosinophils in the host response to cryptococcal infection in the rat.

In contrast, eosinophil recruitment within genetically susceptible mice had detrimental effects for the host. Increased eosinophil recruitment resulted in enhanced IL-5 and led to decreased fungal clearance and damage of lung epithelial cells (Huffnagle et al., 1998). Further to this, surfactant protein D, as described above, promotes the recruitment of eosinophils, through enhancement of IL-5 production (Holmer et al., 2014).

Eosinophils have been shown to be a major source of Th2 cytokine IL-4 during cryptococcal infection. Eosinophil deficient mice infected with *C. neoformans* had fewer Th2 cells and increased Th1/Th17 cells, in addition to a reduction of inflammatory cells (Piehler et al., 2011). This again indicates a detrimental role of eosinophils, whereby a Th2 profile is induced in their presence. An interesting clinical observation of an immunocompetent patient with disseminated *C. neoformans* infection and elevated eosinophil levels, suggests that *Cryptococcus* is able to induce a Th2 profile that may lead to eosinophilia. However, it was noted that this patient had an atypical Th2 profile, and drugs used for treatment may have increased the eosinophil numbers, and this combination may explain the rarity of this observation (Yamaguchi et al., 2008). The potential capacity of cryptococci to change the host towards a Th2 profile may highlight a mechanism of pathogenicity, with an associated detrimental host response of eosinophil recruitment. Here the differences between the rat and mouse models is intriguing and understanding the molecular differences in their immune response would potentially inform cryptococcal pathogenesis generally, as well as the importance of eosinophilia and a predominant Th2 profile in cryptococcal infection, particularly in HIV infected individuals.

## 10 B cell and antibody responses to *Cryptococcus*

Antibodies are generated in response to cryptococcal challenge, with IgM, IgG and IgA each isolated from human patients in descending order of prevalence (Houpt et al., 1994). Antibodies against cryptococci have been found in adults and children (Abadi and Pirofski, 1999; Chen et al., 1999), indicating cryptococcal infection at a young age, and that cryptococcal infection is a common occurrence. Defects in antibody responses are seen in immunocompromised individuals, with B cell repertoire observed in both mouse and human in response to glucuronoxylomannan, a major constituent of cryptococcal capsule, being depleted in HIV infection (Pirofski, 2001). Specific IgM and IgG, but not IgA, against cryptococcal melanin has been observed after mouse infection (Nosanchuk et al., 1998) and immune sera were found to only have high affinity to bind to melanised cryptococci. This is interesting as melanin is considered to be found in the cryptococcal cell wall and suggest that antibodies may have access to the cell wall during infection. Passive immunisation with two different monoclonal IgM antibodies to melanin reduced fungal burden during mouse infection and was able to directly reduce cryptococcal growth *in vitro* (Rosas et al., 2001).

As described above, complement and antibody are required for efficient phagocytosis of *C. neoformans in vitro*, with potential compensation when one opsonin is missing or impaired. In agreement with this the reduced complement mediated phagocytosis caused by increased cryptococcal capsular size is compensated with increase in antibody mediated phagocytosis, demonstrated with C5 deficient mice which have an increased antibody response (Dromer et al., 1989; Zaragoza et al., 2003).

Antibodies have been shown to have a protective effect in lethally infected mice (Fleuridor et al., 1998; Mukherjee et al., 1992), showing that antibodies are effective as opsonins for *C. neoformans*. A mouse model deficient in secreted IgM showed increased death and fungal burden in the blood and brain. Mice with secreted IgM adopted a Th1 profile early during infection and had higher levels of phagocytosis (Marks and Pirofski, 2010). Furthermore, a B1 B cell deficient murine model showed increased fungal burden and decreased alveolar macrophage uptake (through decreased IgM) (Szymczak et al., 2013). This provides an association between B cell responses, and the effector function of phagocytes using antibody for phagocytosis, and suggests that there is requirement for antibody in the normal clearance of cryptococci. Therapy targeting B cell and antibody responses is a potentially fruitful area but further work is needed to understand the mechanisms involved and what defects are present in human disease.

## 11 T cell interactions with cryptococci

The adaptive immune response is unequivocally essential for host eradication of *C. neoformans* with the T cell defects seen in, and so strongly associated with, HIV/AIDS patients (Jarvis and Harrison, 2007). There are several types of T lymphocytes (T cells) involved in the host response to *C. neoformans*, including CD4+, CD8+ and natural killer T (NKT) cells. In addition to T cells, natural killer (NK) cells are lymphoid cells that directly interact with cryptococci. CD4+ T cells are key in orchestrating the type of immune response that results from immune challenge. Naïve CD4+ T cells are activated and differentiate into different subsets, such as Th1, Th2 and Th17, due to the cytokines present. Furthermore, once active CD4+ T cells are able to help B cells, macrophages and CD8+ T cells, to produce antibodies, activate and proliferate respectively (Luckheeram et al., 2012).

Adaptive immune cells are activated when cryptococci are recognised after presentation through antigen presenting cells (APCs). Dendritic cells are likely the primary APC for cryptococci and are able to promote T cell proliferation significantly better than tissue resident alveolar macrophages (Syme and Spurrell, 2002). In addition, eosinophils, but not macrophages, isolated from rats were able to promote CD4+ and CD8+ T cell proliferation through MHC class II and MHC class I molecules respectively (Garro et al., 2010). This eosinophil mediated T cell expansion led to Th1 cytokine production and promoted clearance of fungi (Garro et al., 2010). Within cryptococcal infected CD4+ deficient mice, an increase in CD8+ proliferation is observed, with increased TCR diversity, (Lindell et al., 2006, 2005), indicating that CD8+ are able to function without help from CD4+ cells. Both CD4+ and CD8+ T cells produce pro inflammatory cytokines in response to cryptococcal exposure (Lindell et al., 2006).

Cryptococcal capsular proteins induce a Th2 profile of CD4+ helper cells (Almeida et al., 2001), with this Th2 profile, naïve CD4+ cell activation is abrogated leading to conditions favourable for cryptococcal growth through preventing production of pro inflammatory cytokines. Cryptococcal capsule also may disrupt host T cell responses, through GXM directly inhibiting T cell proliferation, but the mechanism behind this is unclear (Yauch et al., 2006). Similarly, GXMGal is also shown to inhibit proliferation of T cell, potentially through the induction of T cell apoptosis (Pericolini et al., 2006). How cryptococcal capsular modulation of T cell responses is relevant in disease is not clear, as we do not know what the encapsulated state of cryptococci during clearance of infection from the immunocompetent lung. Where this may be very important is the handling of cryptococci in the immunocompromised after CNS invasion where cryptococcal capsule is significantly enlarged (see below for discussion of T cell functionality in cryptococcal meningitis).

CD4+, CD8+ and NK cells can bind directly to cryptococci and act in a fungistatic fashion (Levitz et al., 1994). Cytotoxic CD4+ cells are able to release granulysin, like CD8+ T cells, to kill *C. neoformans* and are more effective at killing cryptococcal cells than CD8+ and NK cells in the blood (Zheng et al., 2007). CD4+ cells also play a role in CD8+ granulysin mediated cryptococcal degradation, whereby granulysin is up regulated and released dependent on CD4+ (Ma et al., 2002).

In addition to CD4+ and CD8+ T cells, there are lymphocytes that can be considered acting as part of the innate immune system involved in the host response to *C. neoformans*: natural killer cells, natural killer T cells, and γδ T cells. Firstly, natural killer cells (NKs) are able release granules, but unlike CD8+ cells, utilise perforin rather than granulysin, through a PI3K-ERK1/2 dependent pathway to cause cryptococcal degradation (Ma et al., 2004; Wiseman et al., 2007). After degranulation the NK cells are still able to continue anti cryptococcal activities, due to constitutive expression of perforin and granulysin, and rearming of NK cells through direct contact with cryptococcal cells (Ma et al., 2004; Marr et al., 2009). Recently, NK cells have also been shown to be key in the immune response to cryptococcal granulomas, having an ability to continue anti fungal degranulation despite an acidic environment (Islam et al., 2013). NK cells are thus important in the clearance of cryptococcal cells and granulomas. Natural killer T cells (NKT) are also able to combat cryptococcal infection. NKT cells are recruited to mouse lungs after infection, and are able to produce IFNγ in response to cryptococcal challenge, resulting in a Th1 profile beneficial for the host to stop cryptococcal infection (Kawakami et al., 2001a,b). In contrast, γδ T cells do not promote fungal clearance in the host response to *C. neoformans*, indicated by mice deficient in γδ T cells that were able to control the fungal infection better than controls, due to a change towards a Th1 profile (Uezu et al., 2004).

## 12 T cell regulated immune balance and macrophage activation: Completing the circle to cryptococcal cell clearance

### 12.1 T helper cell profile

The host T helper subset and associated cytokines are important in the progression and outcome of cryptococcal infection with Th1 and Th17 profiles associated with clearance of cryptococcal infection, and Th2 profiles associated with cryptococcal dissemination and host damage. A preferential Th1/Th17 response in comparison to a Th2 response was shown using an IL 4/IL 13 double knockout murine model. These mice generated a Th1/Th17 response in leukocytes and pulmonary lymph nodes against cryptococcal infection, in comparison to wild type mice that generated a Th2 profile. The Th1/Th17 response was shown to be less damaging to the host through reduced fungal burden, no eosinophilia or airway damage and no systemic IgE load (Zhang et al., 2009). Thus, it appears that generation of a Th2 response from cryptococcal challenge is detrimental to the host, whilst a Th1/Th17 profile is beneficial. Interestingly, in response to IFNγ producing *C. neoformans*, IL 17A is secreted primarily from neutrophils in mice, and may be helpful in the clearance of cryptococcal infection (Wozniak et al., 2011). Similarly, using a murine model, IL 13 a Th2 cytokine, is shown to be detrimental for the host during cryptococcal infection. Overexpressing IL 13 mice were more susceptible to infection, through creation of a Th2 profile whilst inhibiting IL 17 production. IL 33 receptor deficient mice had better survival and reduced fungal burden in comparison to wild types. Using this mouse model, IL-33 was shown to be involved in the induction of type 2 lymphoid cells, alternatively activated macrophages and the associated Th2 profile, leading to a better environment for virulent cryptococci (Flaczyk et al., 2013). Subsequent recruitment of eosinophils and airway inflammation were damaging to the host (Muller et al., 2007). Damage to airways is shown to occur in Th2 profiles and is correlated with production of IgE (Jain et al., 2009). This indicates that Th2 host damage may in part be caused by an allergic response. However, Th1/Th17 profiles are not sufficient to stop cryptococcal dissemination, as IL 14/IL 13 knockout mice, which do not develop Th2 profile, were not protected from dissemination (Zhang et al., 2009). Therefore, at the level of T cell profiling there is a clear and consistent separation in requirements in pro inflammatory pathways. However, greater detail in the positive roles of Th2 responses in resolution of inflammation in cryptococcal infection and the timing of the requirements of the different T helper elements in still required.

### 12.2 What changes in immunocompromisation?

In HIV infection, the cytokine profile changes from Th1 to Th2 over time (Altfeld et al., 2000), and therefore the host immune environment becomes more favourable to cryptococcal infection. HIV affects the determination of the Th1 vs Th2 profile, as both increased viral load and decreased CD4+ cell count lead to prominence of IL4 over IFNγ (Altfeld et al., 2000). Furthermore, CD4+ T cell granulysin mediated killing of cryptococci is not activated in cells from HIV patients (Zheng et al., 2007). A murine model of HIV infected with *Cryptococcus* produced reduced levels of chemokines CCL2 and CCL5, which are immune cell attractants, in comparison to non-infected mice (Leongson and Cousineau-Côté, 2013). Thus, HIV infection may lead to specific innate immune cell recruitment defects as well as general immune imbalance and this will further reduced host capability to combat *Cryptococcus*.

Recently, anti granulocyte macrophage colony stimulating factor (GMCSF) autoantibodies have been implicated as a risk factor for cryptococcal meningitis in HIV negative patients (Rosen et al., 2013). Anti GMCSF antibodies were previously known to be associated with the acquired form of alveolar proteinosis, a disease characterised by an ability to process surfactant in the lung leading to accumulation of material in the alveoli. GMCSF autoantibodies from a non HIV cryptococcal meningitis patient cohort were able to inhibit MIP 1α production in monocytes and the phosphorylation of STAT5. The mechanism of susceptibility of these patients to cryptococcal meningitis is currently unknown but GMCSF is likely to play a role in the recruitment and differentiation of monocytes and DCs during cryptococcal infection and these patients may therefore be unable to activate the secondary responses required for clearance of cryptococci.

Clearance of cryptococcal cells within the central nervous system is possible, and requires Th1 cytokines, in addition to chemokines to attract cells required for degradation (Buchanan and Doyle, 2000; Hill and Aguirre, 1994; Huffnagle and McNeil, 1999). The immune phenotype observed in cryptococcal meningitis patients is not well understood. A recent study by Jarvis and colleagues has begun to address this area: By measuring both systemic and CNS cytokines and CD4+ responses, and comparing to *M. tuberculosis* and cytomegalovirus responses in the same samples. Using their method, the specificity and limitations of the immune response to CM could be delineated and associated with survival (Jarvis, seminar presentation 10.1, ICCC9; Jarvis et al., 2013). CD4+ cells specific to cryptococcal antigen showed little polyfunctionality and were dominated by MIP 1α producing cells with smaller but equal groups of IFNγ and TNFα producing cells. Surviving patients had higher proportions of IFNγ and TNFα producing cells. In contrast to animal models of cryptococcosis no imbalance of Th1 and Th2 responses was observed but rather it is the lack of Th1 responses that was more common with an unfavourable prognosis (Jarvis et al., 2013). However, it should be noted that Th2 responses should not always be categorised, as being detrimental or unnecessary in handling cryptococcal infection. Changes in polarisation are seen in mouse pulmonary models, where the M1 and M2 balance shifts over time (Davis et al., 2013). Therefore, Th2 responses may have important roles at different stages of human infection e.g. the resolution of inflammation after clearance of infection.

### 12.3 Eradicating cryptococci

Just as macrophages, and subsequently dendritic cells, will initiate adaptive responses principally through T lymphocytes, the primary purpose of the T cell response is to eradicate cryptococci through generation of a suitably antimicrobial environment. As stated above, in the lung this is almost certainly normally through a resolving granuloma. In the sites of disseminated infection and, most importantly, the CNS in cryptococcal meningitis the direct effectors in clearance are yet to be proven.

Macrophages activated either with Th1/Th17 cytokines or Th2 cytokines had different interactions with cryptococci. A Th1/Th17 profile was shown to activate macrophage anti cryptococcal activities, causing a reduction of fungal proliferation (Voelz et al., 2009), thus macrophages require a Th1/Th17 profile to function efficiently. Furthermore, classically activated macrophages are important in the clearance of *C. neoformans*, whereas alternatively activated macrophages may play a role in the pathogenicity of cryptococci. Th1 profiles classically active macrophages whereas Th2 cytokines lead to alternatively activated macrophages (Arora et al., 2011), thus a Th1 profile may lead to the correct type of subsequent macrophage activation, again suggesting Th1 profile is best to eradicate *C. neoformans*. The classically activated macrophages and dendritic cell lineages acting directly to eradicate cryptococci down stream of Th1 CD4+ responses are currently our most likely candidates (Hardison et al., 2012, 2010).

It is difficult to measure the direct anti cryptococcal activity of effector cells *in vivo* (Hardison et al., 2012). *In vitro* assessment showed a small magnitude change in fungicidal activity but it is likely that *ex vivo* isolation has a large impact on their phenotype and therefore fungicidal effectiveness. Interestingly, the classically activated macrophage phagosome combination of low phagosomal pH and nitric oxide (NO) in a reconstituted *in vitro* system showed a good ability in controlling cryptococcal proliferation (Alspaugh and Granger, 1991). A recent study gives very good support to this idea; STAT 1 deficient mice showed an inability to generate a strong Th1 profile and to produce classically activated M1 macrophages (Leopold Wager et al., poster presentation 121, ICCC9; Leopold Wager et al., 2014). Most interestingly the anti fungal activity defect phenotype was associated with severely reduced NO production from the macrophages isolated from the lungs of infected mice.

## 13 The immune response to *C. gattii*

As the major disease causing organism most studies have focussed on *C. neoformans*. We know that *C. gattii* is able to infect those with apparently normal immune systems suggesting that there must be large differences in the immune response to this species. One intriguing difference is that *C. gattii* generally has a reduced ability to grow within macrophages than *C. neoformans* (Voelz et al., 2014). The exception is the hyper proliferative genotypes isolated from the Vancouver Island Outbreak (VIO; (Ma et al., 2009)) and elsewhere in North America (Byrnes et al., 2010; Springer et al., 2014). These hyper proliferative strains show changes in mitochondrial gene expression and have a tubular mitochondria phenotype that closely correlates with macrophage parasitism and virulence in a mouse model (Ma et al., 2009). Recently, this has been shown to be dependent on the reactive oxygen species (ROS) response of host macrophages (May and Voelz, seminar presentation 8.4, ICCC9; Voelz et al., 2014). Inhibition of ROS production in macrophages was sufficient to block the increased intracellular proliferation of the VIO strains i.e. a host antimicrobial response is required for maximal virulence of the pathogen. How these strains are better able to proliferate in macrophages is unclear but this is a phenomenon that appears to function through modulating the host phagosome, since coinfection of hyper and hypo proliferative strains together leads to a hyper proliferation phenotype conferred to both infecting strains.

The primary immune response to *C. gattii* is still unclear with both a hyper and hypo inflammatory response reported and there is some evidence from mouse models that *C. gattii* may more readily cause pulmonary infection than spread to the CNS (Ngamskulrungroj et al., 2012). *In vitro* stimulation of monocytes demonstrated higher levels of inflammatory cytokines, while the pulmonary immune response to a VIO strain was poor in comparison to a non VIO and to a *C. neoformans* strain when assessed by histology (Okubo et al., 2013). Fewer Th1/Th17 lymphocytes are present in the lungs of *C. gattii* infected mice (Angkasekwinai et al., 2014) and there is reduced levels neutrophil infiltration in comparison to *C. neoformans* infection (Cheng et al., 2009). However, immunisation with *C. gattii* cell wall or cytoplasmic components can confer a protective response to subsequent *C. gattii* infection (Chaturvedi et al., 2014).

Mice deficient in C1q or C3 or depletion of complement with cobra venom factor showed overwhelming lung fungal burden after infection with *C. gattii* (Mershon et al., 2009). *C. gattii* is a able to disrupt dendritic cell maturation by blocking TNFα release (Huston et al., 2013). In this case DCs are not activated and this leads to subsequent non activation of further TNFα secretion, antigen presentation and reduced T lymphocyte proliferation. This is further supported with TNFα induction or addition of exogenous TNFα restoring DC functions. The resulting absence of adaptive response may therefore enable infection within immune competent hosts by *C. gattii* (Huston et al., 2013). Finally, it is important to note that there may be cryptic immune pathology in those that are infected with *C. gattii*. A recent study has also identified GMCSF autoantibodies as a risk factor for *C. gattii* and this highlights that further work is needed to understand differences in both host and pathogen in *C. gattii* infection (Kwon-Chung et al., seminar presentation 5.1, ICCC9; Saijo et al., 2014).

## 14 Conclusions and perspectives

The intricacy and complexity of the immune response to cryptococci is perhaps, on first glance, surprising. As cryptococci are not obligate human pathogens (or even obligate animal pathogens) and there is no clear evidence that *Cryptococcus* has evolved to be an animal pathogen. However, *Cryptococcus* has evolved to exploit an environmental niche (with its own predator prey relationships) that has driven survival traits that allow it to thrive in its niche that also give it advantages in the human body (Bliska and Casadevall, 2009; Steenbergen and Casadevall, 2003). Furthermore, land animals have been exposed to a wide variety of potentially lethal microorganism via their respiratory systems for millions of years and we have evolved many different and interacting defences against organisms such as *Cryptococcus*. This constitutes an arms race; one that we are winning as, although disease burden and mortality due to respiratory and opportunistic infection are very high, exposure and subclinical infection must be several orders of magnitude greater.

Where we are losing, and where cryptococcal disease burden is centred, is in individuals with compromised immune systems. Evidence from all areas, clinical, animal models and *in vitro* experiments, points to the central importance of the macrophage/T cell axis. Immune defects in T cell development (blood proliferative disorders), numbers and functionality (HIV infection, immunosuppressant treatment) and signalling (antibodies to TNFα) all lead to increased risk of disseminated cryptococcal infection.

As we have described there are many examples of how cryptococci may be killed by host immune cells but it is still not clear what the critical mechanism is during clearance of infection and attempts to recapitulate this *in vitro* have not been conclusive. Most likely it will be a combination of mechanism that may be thought of as a series of barriers to disseminated infection, ones that are extremely rarely breached except in the case of significant defects in immunity. However, if immune therapy as an adjunct to antifungal treatment is to be a reality we need to understand the precise effector pathways that are required.

Animal models have been central to our understanding of the required immune response to cryptococcal infection for clearance and what defects dispose to disseminated infection. Clinical data has been extremely helpful in placing this understanding in the context of human disease but there is much to be done in this challenging area. Increasing knowledge of immunity late in infection would allow us to better manage both the clearance of the large CNS fungal burden and related pathology both during and after antifungal treatment.
